# Associations of reallocating sedentary leisure-time to alternative discretionary movement behaviours with incident cardiometabolic diseases in 0.5 million Chinese adults

**DOI:** 10.1016/j.lanwpc.2025.101524

**Published:** 2025-03-23

**Authors:** Paul J. Collings, Mengyao Wang, Harrison Hin Sheung Ho, Shiu Lun Au Yeung, Parco M. Siu, Benjamin J. Cowling, Stephen J. Sharp, Søren Brage, Nicholas J. Wareham, Huaidong Du, Derrick A. Bennett, Youngwon Kim, China Kadoorie Biobank (CKB) Study Collaborative Group

**Affiliations:** a School of Public Health, Li Ka Shing Faculty of Medicine, University of Hong Kong, Hong Kong Special Administrative Region of China; b MRC Epidemiology Unit, School of Clinical Medicine, University of Cambridge, Cambridge, UK; c Nuffield Department of Population Health (NDPH), University of Oxford, Oxford, UK

**Keywords:** Sedentary behaviour, Sleep, Physical activity, Isotemporal substitution, Cardiovascular diseases, Prospective study

## Abstract

**Background:**

In Chinese adults, there is a considerable burden of sedentary behaviour. This study aimed to estimate the implications of reallocating sedentary leisure-time to non-sedentary behaviours for incident cardiometabolic diseases.

**Methods:**

A prospective cohort study of 462,370 Chinese adults (mean age 51 years; 59% female) who were free from diabetes and cardiovascular diseases at baseline. Isotemporal substitution Cox regression models were used to estimate the associations of reallocating self-reported sedentary leisure-time to the same amount of sleep, housework, Taichi, or conventional exercise (e.g., walking, jogging, ball games, swimming) with the risk of incident diabetes, stroke, and myocardial infarction (MI). The results are reported as adjusted hazard ratios and 95% confidence intervals per 30 min/day time exchanges. Potential impact fractions were calculated to estimate the proportional reductions in incident disease cases associated with time substitutions, assuming causality.

**Findings:**

During >5.25 million person-years of follow-up, 19,738 incident diabetes, 51,460 stroke, and 6767 MI cases were accrued. Lower disease risks were found for replacement of sedentary leisure-time by sleep (diabetes: 0.97 [0.95–0.99], stroke: 0.98 [0.97–0.99], MI: 0.97 [0.94–0.99]; in participants who slept <7 h/day), housework (diabetes: 0.97 [0.97–0.98], stroke: 0.99 [0.98–0.99], MI: 0.97 [0.95–0.98]), Taichi (diabetes: 0.97 [0.95–0.99], stroke: 0.98 [0.97–0.99], MI: 0.95 [0.92–0.98]), or conventional exercise (diabetes: 0.97 [0.95–0.99], stroke: 0.97 [0.95–0.98], MI: 0.92 [0.88–0.96]). Potential impact fractions ranged from an estimated 3.5% (95% confidence interval: 3.1–3.9%) fewer cases of incident stroke when replacing sedentary leisure-time with housework, to an estimated 9.6% (5.9–13.3%) fewer cases of incident MI when reallocating sedentary leisure-time to conventional exercise.

**Interpretation:**

Replacing sedentary leisure-time with behaviours such as housework, Taichi, sleep (in short sleepers) and conventional exercise is associated with lower risks of common cardiometabolic diseases in Chinese adults. Prevention strategies should be developed to promote movement behaviours and optimal levels of sleep at the expense of sedentary leisure-time.

**Funding:**

This analysis was supported by a 10.13039/501100005847Health and Medical Research Fund (HMRF) Research Fellowship (grant no: 06200087).

**Research in context**

**Evidence before this study**

Interventions have demonstrated that sedentary leisure-time (i.e., non-occupational seated activities such as TV viewing, reading, playing cards, using a computer outside of work) can be effectively reduced in adults. It should be considered that any potential health benefit arising from reduced sedentary leisure-time likely depends on whether the resultant time void is filled with sleep or different discretionary physical activities. Isotemporal substitution methods have been developed to model theoretical switching between behaviours. We performed a literature search using PubMed on January 16, 2025, incorporating terms related to sedentary time, physical activity, sleep, isotemporal substitution, diabetes and cardiovascular diseases; the search yielded 26 original studies. In general, whilst beneficial associations have been reported for sleep and light physical activity, the strongest association with cardiometabolic health outcomes have been observed when time is reallocated from sedentary behaviour to exercise of at least moderate intensity. Studies performed in Western populations dominate, and the evidence primarily comprises small cross-sectional investigations of cardiometabolic biomarkers. It is also a weakness that most studies have not included sleep as a component of time-use; non-isotemporal analyses have shown U-shaped associations between sleep duration with the risk of cardiovascular diseases.

**Added value of this study**

We investigated the potential for replacing sedentary leisure-time with alternative movement behaviours or sleep in the prevention of diabetes, stroke, and myocardial infarction in an ongoing population-based cohort of ⁓0.5 million Chinese adults with a median follow-up of 12 years. We focussed on sedentary leisure-time because it may be easier to modify than time spent sedentary as part of travel or work, and investigated diverse replacement behaviours within the home and leisure domains. To account for potential non-linear associations with outcomes, sleep was incorporated as a piecewise variable. We found that reallocating sedentary leisure-time to behaviours including sleep (in short sleepers), housework, Taichi, and conventional exercise is associated with lower risks of common cardiometabolic diseases in Chinese adults.

**Implications of all the available evidence**

Our findings contribute to emerging evidence about the potential role of less intense physical behaviours for the prevention of cardiometabolic diseases, whilst reinforcing the importance of engaging in physical activity of at least moderate intensity. Promoting gentle forms exercise and non-exercise behaviours (e.g., housework, sleep) may be an important behaviour change strategy for adults who are initially unable to replace sedentary time with more structured activities and higher-intensity forms of exercise.

## Introduction

Cardiometabolic diseases, such as diabetes and cardiovascular disorders, constitute a major threat to public health.^1^ Over the past decades the prevalence of diabetes has increased rapidly in China, rising from <1% in 1980 to 3.7% in 1990, and reaching almost 7% by 2016.^2^^,^^3^ It is estimated that 140.9 million individuals in China currently have diabetes (equivalent to 13% prevalence and one-quarter of all cases worldwide) and this number is expected to exceed 174 million by 2045.^4^ From 1990 to 2016 the prevalence of cardiovascular diseases in the country also doubled to reach nearly 94 million cases.^5^ Recent evidence indicates that cardiovascular diseases account for more than 40% of all deaths in China, and that mortality from serious cardiovascular events, such as stroke and myocardial infarction (MI), has continued to increase over time.^2^

The accelerated chronic disease burden in China has coincided with a societal transition to reduced physical activity and increased sedentariness.^6^^,^^7^ Sedentary behaviour comprises all accumulated time in waking activities that are characterised by low energy expenditure and which are performed in a sitting, reclining or lying posture.^8^ Sedentary leisure-time has most markedly increased over time,^7^ with Chinese adults engaging in more than 3 h/day of TV viewing, reading, playing cards or mahjong, and using a computer outside of work.^9^ The predominant leisure-time sedentary behaviour is TV viewing^10^^,^^11^ which is strongly and consistently associated with increased risks of diabetes and cardiovascular diseases.^12^^,^^13^ In response to the physical inactivity crisis, the Chinese government published new physical activity guidelines recommending lower sedentary time,^14^ and promoted regular exercise of at least moderate intensity as part of its *Healthy China 2030* action plan.^15^^,^^16^ These are important objectives, but they do not fully reflect global guidance that replacing sedentary time with any physical activity likely provides health benefits,^17^ primarily because there is limited evidence specific to the Chinese population. Additional studies are required to inform future Chinese physical behaviour guidelines and action plans.

Intervention studies have demonstrated that sedentary leisure-time can be effectively reduced in adults,^18^ It is important to consider, however, that any potential health benefit arising from reduced sedentary leisure-time likely depends on whether the resultant time void is filled with sleep or different discretionary physical activities. Isotemporal substitution methods have been developed to model theoretical switching between movement behaviours.^19^ These methods quantify, for example, the potential health implications of substituting a fixed amount of sedentary leisure-time with exercise or non-exercise behaviours. To our knowledge, only one study has incorporated isotemporal substitution methodology to investigate the associations of reallocating sedentary time to physical activity on the risk of incident cardiovascular diseases in Chinese adults.^20^ That study focussed on total sedentary time, encompassing sedentary time spent across transport and work-related domains, which may be less amenable to change than behaviours during leisure-time. Furthermore, sleep duration was not included as a component of overall time-use. It is estimated that nearly one-quarter of Chinese adults fail to sleep for the minimum recommended 7 h/day,^21^ which is associated with a higher risk of diabetes and cardiovascular events.^22^, ^23^, ^24^ Incorporating sleep duration as part of time-use compositions could yield findings that are highly relevant to public health.

There is currently no consensus among public health authorities about the optimal ‘balance’ of movement behaviours or the types of physical activities that are most beneficial for Chinese adults.^25^ The aim of this study was to quantify the prospective associations of theoretically reallocating sedentary leisure-time to different behaviours with key cardiometabolic diseases in a large sample of Chinese adults.

## Methods

### Study population

This study used data from the China Kadoorie Biobank (CKB) a large-scale prospective cohort of half a million Chinese adults.^26^ In brief, between June 2004 and July 2008, residents aged 35–74 years from 10 regions in China were invited to attend research clinics (*n* = 1,801,200). The study areas were selected to cover a wide range of disease patterns and to maximise socioeconomic diversity, rather than to represent the general population in China. Nearly one-third of residents in the target age range consented to take part (*n* = 499,432). To encourage participation, individuals who attended the baseline survey and who were just outside the target age range were not turned away (*n* = 13,292), making the actual baseline age range 30–79 years (*n* = 512,724).

**Fig. 1:**
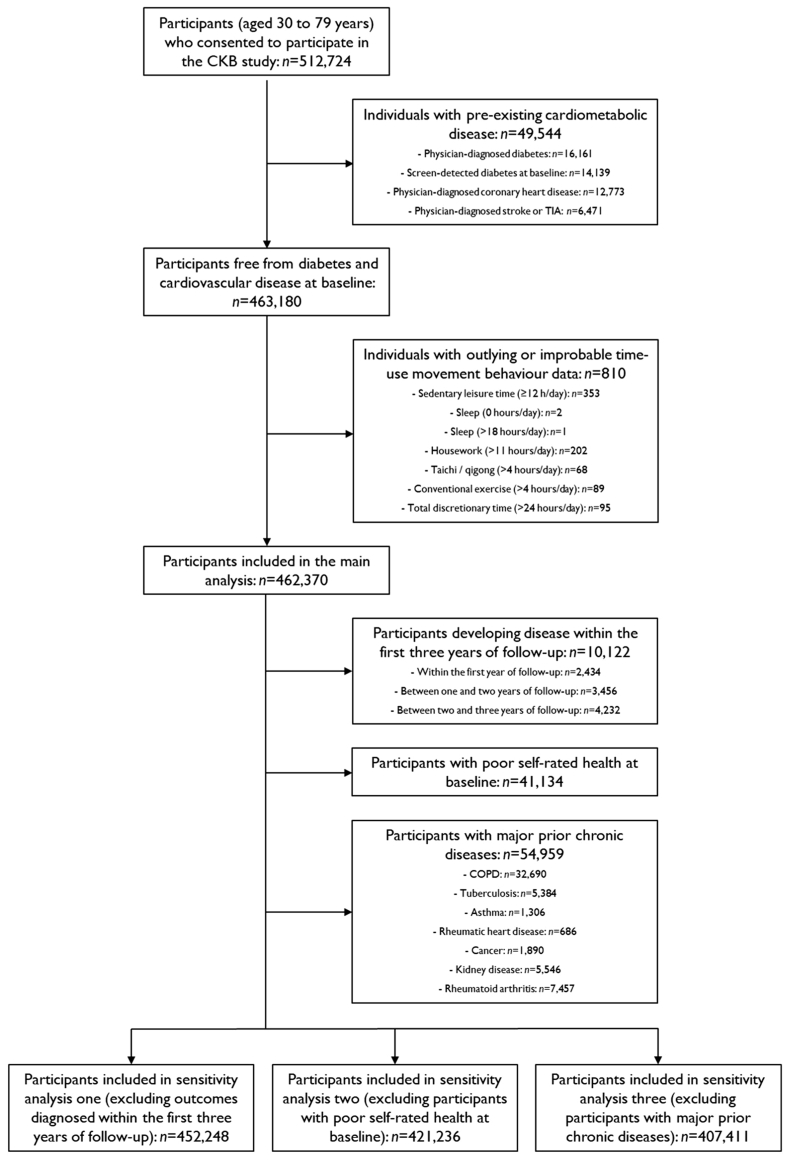
**Participant flow diagram**. Abbreviations: CKB, China Kadoorie Biobank; COPD, Chronic obstructive pulmonary disease; TIA, Transient ischaemic attack.

Trained health workers performed the collection of baseline demographic, lifestyle and general health information via a computerised questionnaire. A 10 mL non-fasting blood sample was collected and used to assess random plasma glucose (RPG) levels; in the event of an elevated reading (i.e., RPG between 7.1 and 11.0 mmol/L) but without prior diagnosis of diabetes, fasting glucose levels were measured the next day. Participants with screen-detected diabetes (i.e., RPG ≥11.1 mmol/L or fasting glucose ≥7.8 mmol/L) at baseline were excluded from this analysis (*n* = 14,139), as were participants with prior physician-diagnosed diabetes, coronary heart disease, stroke or TIA (*n* = 35,405), and participants with outlying or improbable movement behaviour data (*n* = 810). A total of 462,370 participants (90.3% of the starting sample) were included in the analysis (Fig. 1). Ethical approval was obtained from the China Center for Disease Control and Prevention (Beijing, China, approval number: 005/2004, 9.7.2004) and the Oxford Tropical Research Ethics Committee, University of Oxford (UK, approval number: 025−04, 3.2.2005). All participants provided written informed consent before participation.

### Role of the funding source

The sponsor had no role in the study design, data analysis, data interpretation, or writing of the manuscript. The corresponding author had full access to the data and had final responsibility for the decision to submit for publication.

### Movement behaviours

Information about sedentary leisure-time and discretionary physical activities performed in the past 12 months was collected using questions that were adapted from validated instruments,^27^^,^^28^ with some modifications after a CKB pilot. Participants reported the number of hours per week they had spent in sitting activities during leisure-time (e.g., TV viewing, reading, playing cards or mahjong, and using a computer outside of work) and doing housework. They also reported how often they had exercised for leisure, the main type of exercise performed, and the number of hours per week they usually performed that type of exercise. The data were used to derive the average daily amount of leisure-time spent performing Taichi (including qigong) or conventional exercise (walking/jogging or aerobic exercise/swimming/ball games/other). Participants also reported the number of hours they had typically slept per day, inclusive of naps. Sleep duration has been reported to exhibit a U-shaped relationship with cardiometabolic diseases in the CKB cohort.^29^ For this reason, to satisfy the isotemporal substitution modelling assumption of linearity between independent and dependent variables, sleep duration was incorporated as a piecewise variable with a breakpoint at 7 h/day (<7 h/day and ≥7 h/day; each variable showed approximately linear associations with outcomes).^30^^,^^31^ For the analysis, all movement behaviour data were converted to minutes per day (minutes/day).

### Ascertainment of cardiometabolic diseases

Incident disease cases were identified via electronic linkage to local mortality records, chronic disease registries, and the national health insurance claim system which has almost universal (approximately 99%) coverage across all study areas. The primary outcomes of the present study were defined according to the International Classification of Diseases Tenth Revision (ICD-10) taxonomy: diabetes (E10-14), MI (I21-23), and stroke (I60–I61 & I63–I64). Trained staff who were blinded to baseline participant information coded all cases. Positive predictive values in the CKB cohort are high for new-onset diabetes (97%), stroke (91%), and ischaemic heart disease (88%).^32^^,^^33^ Follow-up was censored on January 1st 2019, by which time 43,581 participants (9.4%) had died and only 3675 (0.8%) were lost to follow-up. Incident disease cases were identified as the first diagnosis of each respective outcome over a median follow-up period of approximately 12 (interquartile range: 11–13) years.

### Potential confounders

We considered the following variables to be potential confounders as they are plausibly associated with exposures and outcomes. Demographic factors included sex (male/female), recruitment location (ten regions: five rural and five urban), educational attainment (below middle school/middle or high school/college or university), and total household income in the last year (<10,000/10,000–34,999/≥35,000 yuan). Participants self-reported their work-related physical activity (mainly sedentary or standing/manual [including heavy manual and rural farming]/not in paid employment [including unemployed, homemaker, disabled and unable to work]), smoking status (never/occasional or former/regular smoker [smokes most days if not daily]), frequency of alcohol consumption in the past 12 months (never regular/occasional or ex-regular/regular drinker [consumes alcohol at least monthly]), and whether they usually napped during the day (usually/usually in summer/no). A validated questionnaire was used to capture how frequently in the past 12 months (never or rarely/monthly/1–3 days per week/4–6 days per week/daily) participants had consumed 12 major food groups. Five food groups, each shown to exhibit associations with cardiometabolic diseases in the CKB cohort,^34^, ^35^, ^36^, ^37^, ^38^ were used to create a healthy diet score. To calculate the score, more frequent consumption of fresh fruit, coarse grains, and eggs were assigned ascending values (from one to five), more frequent consumption of preserved vegetables and red meat were assigned descending values (from five to one), and all values were summed. The healthy diet score ranged from five to twenty-five, with higher values indicating a healthier diet. Participants reported if they were taking any antihypertensive medications (no/yes) and if they had a family history of diabetes or cardiovascular disease (in parents or siblings: no/yes).

### Statistical analysis

#### Isotemporal substitutions

Cox regression models with age as the underlying timescale were initially used to estimate the associations of sedentary leisure-time and each of the alternative discretionary movement behaviours with incident diabetes, MI, and stroke. The linearity of associations was assessed by fitting cubic spline regression models with five knots. There were no substantial deviations from linearity; hence, all time-use components were subsequently modelled linearly as continuous variables. Isotemporal substitution Cox regression models, stratified by sex and region and adjusted for all potential confounders, were subsequently used to estimate the prospective associations of reallocating sedentary leisure-time to equivalent sleep, housework, Taichi, or conventional exercise with incident cardiometabolic diseases.^39^ The models were adjusted for total reported discretionary time (the sum of discretionary movement behaviours) and omitted sedentary leisure-time from the linear predictor. The results represent the potential implications for each outcome of exchanging sedentary leisure-time with equivalent time in other movement behaviours. The main results are reported as hazard ratios and 95% confidence intervals, per 30 min/day of time exchanged. We also present the results for up to 60 min/day of time exchanged, expressed in 10-min increments. The proportional hazards assumption for each covariate was met based on Schoenfeld residuals and log–log plots.^40^

#### Effect modification

Participant sex (male/female), baseline age group (30–50/50–60/60–80 years), central obesity status (no/yes [waist circumferences ≥90 and ≥85 cm for men and women, respectively]^41^), and antihypertensive medication use (no/yes) were investigated as potential effect modifiers, by using likelihood ratio tests that compared models with and without the relevant interaction terms (e.g., time-substitution parameter∗sex). Stratified analyses were performed to quantify time substitution results within subgroup strata.

#### Potential impact fractions

To examine the potential public health effect of time-substitution estimates, we calculated potential impact fractions for each outcome by using the ‘distribution shift’ method, based on a normal distribution for sedentary leisure-time.^42^^,^^43^ The results provide an estimate of the proportion of disease cases that could potentially have been averted in the CKB cohort, if sedentary leisure-time was reduced by 30 min/day and exchanged for a 30 min/day increase in an alternative discretionary behaviour, assuming causality of associations. Confidence intervals were obtained via bootstrap sampling with 1000 replications.

#### Sensitivity analysis

To determine the robustness of the time-substitution associations we excluded from analyses: 1) incident disease cases accrued within the first three years of follow-up, 2) participants with poor self-rated health at baseline, and 3) participants with any self-reported prior disease at baseline (emphysema or bronchitis, tuberculosis, asthma, rheumatic heart disease, cancer, kidney disease, rheumatoid arthritis). We estimated time-substitution associations by sequentially adjusting for potential confounders and waist circumference. All analyses were performed in Stata/MP 17.^44^

## Results

### Descriptive characteristics

During >5.25 million person-years of follow-up there were 19,738 incident diabetes, 51,460 stroke, and 6767 MI cases. Table 1 provides a comprehensive summary of participant characteristics. In brief, the mean (± standard deviation) age at baseline was 51.2 (±10.5) years, the majority (59%) of participants were female, and approximately one-fifth (22.2%) of participants met the criteria for central adiposity. Compared with the lowest category of sedentary leisure-time, a larger proportion of participants with higher sedentary leisure-time were: male, higher educated, from higher income households, not in paid employment, regular smokers and drinkers, using antihypertensives, and had a family history of diabetes, heart attack or stroke. Table 2 shows that sedentary leisure-time accounted for nearly one-quarter of all reported discretionary time. Most participants (80.4%) reported that they had never or rarely performed exercise in the last 12 months, and approximately one-fifth (22.3%) reported sleeping less than the minimum recommended 7 h/day.

**Table 1:** Descriptive characteristics for the whole sample and by categories of sedentary leisure-time.

	Whole sample (*n* = 462,370)	Sedentary leisure-time (minutes/day)				
		≤60 (*n* = 58,574)	>60–≤120 (*n* = 111,074)	>120–≤180 (*n* = 137,654)	>180–≤240 (*n* = 78,528)	>240 (*n* = 76,540)
Female (%)	59.0	68.2	60.6	56.2	56.3	57.3
Age (years)	51.2 ± 10.5	52.4 ± 11.2	51.2 ± 10.3	50.7 ± 10.3	50.8 ± 10.5	51.7 ± 11.1
Education (%)						
Below middle school	79.2	81.8	79.9	78.7	78.0	78.7
Middle or high school	15.1	13.5	14.7	15.4	15.9	15.4
College or university	5.7	4.7	5.4	5.9	6.2	5.9
Annual household income						
<10,000 yuan	28.6	33.8	29.7	27.8	27.4	26.6
10,000–34,999 yuan	53.4	52.0	52.9	53.1	53.1	53.1
≥35,000 yuan	18.0	14.1	17.4	19.1	19.5	20.3
Work-related activity						
Sedentary or standing	19.2	24.2	21.5	19.3	16.6	11.6
Manual	54.5	55.2	55.3	55.2	54.5	51.3
Not in paid employment	26.3	20.6	23.2	25.5	28.9	37.1
Smoking status (%)						
Never	62.1	63.6	63.3	62.5	61.7	61.2
Occasional or former	12.4	12.6	12.5	12.3	12.0	11.9
Regular smoker	25.5	23.8	24.1	25.3	26.3	26.9
Alcohol intake (%)						
Infrequent	45.3	48.0	46.2	45.0	43.5	43.7
Occasional or ex-regular	35.5	34.6	35.2	35.6	36.1	36.0
Regular drinker	19.2	17.4	18.5	19.4	20.3	20.2
Daytime napping						
Usually	19.6	18.1	20.0	21.1	21.0	19.3
Usually in summer	40.9	39.3	40.4	40.9	40.8	40.0
No	39.5	42.6	39.7	38.0	38.2	40.7
Healthy diet score	14.0 ± 2.8	13.7 ± 2.2	13.9 ± 2.1	14.0 ± 2.1	14.1 ± 2.2	14.1 ± 2.3
Treated hypertension (%)	7.0	5.9	6.7	7.3	7.5	7.6
Family history of disease (%)	24.5	23.2	23.9	24.5	25.4	25.2
Waist circumference (cm)	79.7 ± 9.6	78.5 ± 9.7	79.2 ± 9.1	79.8 ± 9.2	80.3 ± 9.3	80.8 ± 9.6

Values are proportions or mean ± standard deviation calculated from logistic or linear regressions adjusted for sex, age and region as appropriate.

Abbreviation: MI, Myocardial Infarction.

**Table 2:** Summary of discretionary movement behaviours.

	Time (minutes/day)	Proportion of total discretionary time (%)
Sedentary leisure-time	179.4 ± 90.4	23.7
Sleep duration	444.3 ± 81.3	58.6
Housework	122.6 ± 89.4	16.2
Taichi/qigong	7.7 ± 24.7	1.0
Conventional exercise	3.8 ± 18.1	0.5

Absolute values are mean ± standard deviation. Total discretionary time constitutes the sum of all movement behaviours (mean ± standard deviation: 757.8 ± 152.5 min/day). Participants who reported exercising in their leisure-time at least 1–2 times/week over the last 12 months (*n* = 90,616) stated their main exercise type was Taichi/qigong (65.8%), walking (13.1%), jogging or aerobic exercise (8.2%), ball games (5.9%), other (5.8%), and swimming (1.2%).

### Isotemporal substitutions

**Fig. 2:**
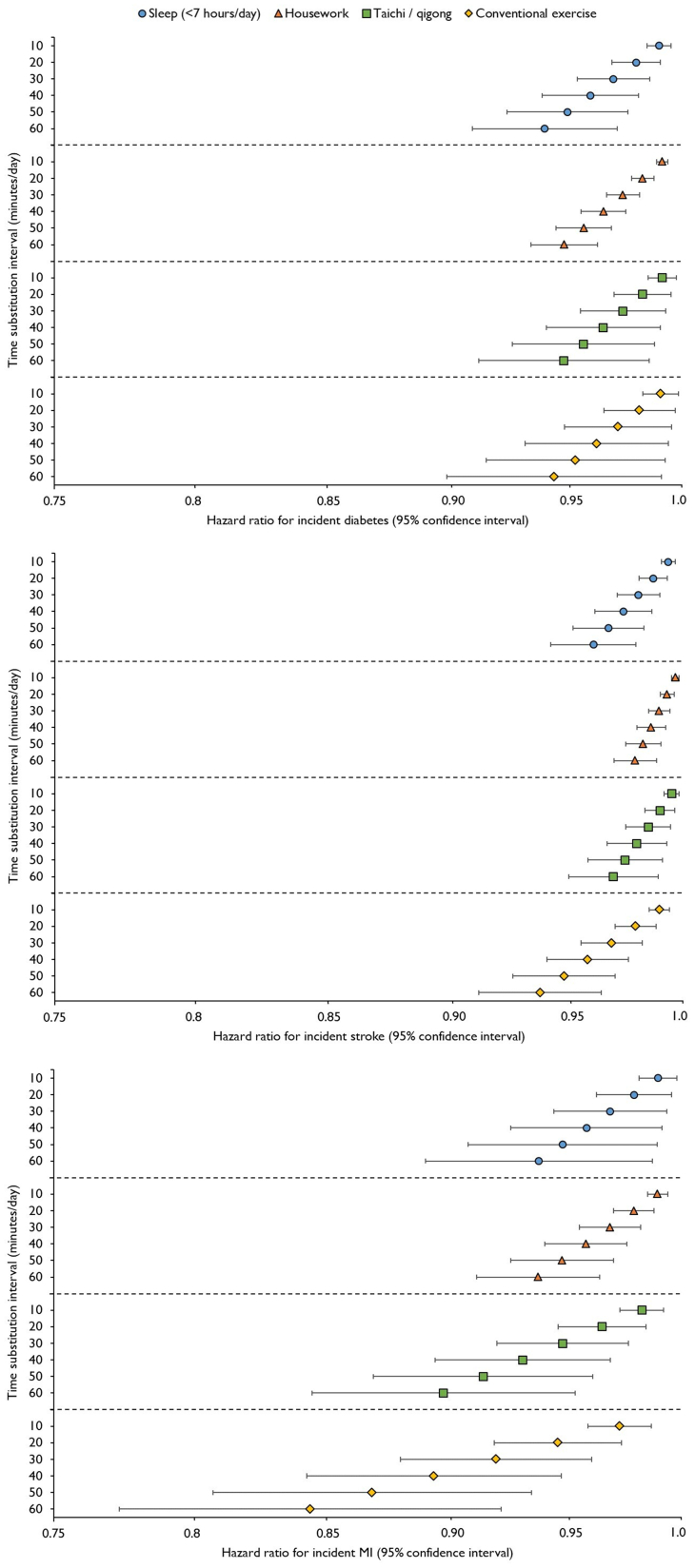
**Associations of reallocating sedentary leisure-time to alternative discretionary movement behaviours with incident cardiometabolic diseases, in 10 min/day time-substitution increments (log scale)**. Values calculated from Cox regression models, using age as the underlying timescale, and incorporating sex and region strata. Models adjusted for educational attainment, household income, work-related activity, smoking status, alcohol intake, daytime napping, healthy diet score, use of antihypertensive medication, family history of diabetes, heart attack or stroke, and total discretionary time-use. The results for reallocating sedentary leisure-time to sleep in longer (≥7 h/day) sleepers are shown in Table S1. Abbreviation: MI, Myocardial Infarction.

Table 3 shows that reallocating sedentary leisure-time to each of the alternative discretionary behaviours (except to sleep in those that reported sleeping ≥7 h/day) was inversely associated with all cardiometabolic disease outcomes. Irrespective of the replacement behaviour, displacing 30 min/day of sedentary leisure-time was associated with 3% lower risk of diabetes (for instance, the hazard ratio [95% confidence interval] associated with reallocating sedentary leisure-time to housework was 0.97 [0.97–0.98]). In short (<7 h/day) sleepers, replacing 30 min/day of sedentary leisure-time with sleep was associated with 2% (0.98 [0.97–0.99]) and 3% (0.97 [0.94–0.99]) lower risk of stroke and MI, respectively. Shifting 30 min/day of sedentary leisure-time to housework was associated with 1% (0.99 [0.98–0.99]) and 3% (0.97 (0.95–0.98)) lower risk of stroke and MI, and equivalent replacement of sedentary leisure-time with Taichi was associated with 2% (0.98 (0.97–0.99)) and 5% (0.95 (0.92–0.98)) lower risk of the same outcomes. The strongest associations were observed when sedentary leisure-time was exchanged for conventional exercise. Reallocating 30 min/day of sedentary leisure-time to conventional exercise was associated with 3% (0.97 (0.95–0.98)) and 8% (0.92 (0.88–0.96)) lower risk of stroke and MI, respectively. Fig. 2 illustrates time-substitution associations expressed in 10-min intervals. Reallocating as little as 10 min/day of sedentary leisure-time to conventional exercise was associated with approximately 1% (0.99 (0.98–0.99)) and 3% (0.97 (0.96–0.99)) lower risk of stroke and MI, respectively, whilst substituting 60 min/day was associated with 6% (0.94 (0.91–0.96)) and 16% (0.84 (0.77–0.92)) lower risk of the same outcomes. To achieve comparable risk reductions, reallocating at least twice as much sedentary leisure-time to each of the alternative discretionary movement behaviours may be necessary. Table S1 provides full results of the incremental time-substitution analysis.

**Table 3:** Associations of reallocating sedentary leisure-time to alternative discretionary movement behaviours with incident cardiometabolic diseases, per 30 min/day time exchanges.

	Incident diabetes (*n* = 19,738)	Incident stroke (*n* = 51,460)	Incident MI (*n* = 6767)
Sleep			
Short sleepers (<7 h/day)	0.97 (0.95–0.99)	0.98 (0.97–0.99)	0.97 (0.94–0.99)
Longer sleepers (≥7 h/day)	1.00 (0.99–1.01)	1.00 (1.00–1.01)	0.99 (0.98–1.01)
Housework	0.97 (0.97–0.98)	0.99 (0.98–0.99)	0.97 (0.95–0.98)
Taichi/qigong	0.97 (0.95–0.99)	0.98 (0.97–0.99)	0.95 (0.92–0.98)
Conventional exercise	0.97 (0.95–0.99)	0.97 (0.95–0.98)	0.92 (0.88–0.96)

Values are hazard ratios (95% confidence interval) calculated from Cox regression models, using age as the underlying timescale, and incorporating sex and region strata. Models adjusted for educational attainment, household income, work-related activity, smoking status, alcohol intake, daytime napping, healthy diet score, use of antihypertensive medication, family history of diabetes, heart attack or stroke, and total discretionary time-use.

Abbreviation: MI, Myocardial Infarction.

### Associations in population subgroups

Table S2 provides results of the interaction analysis between sedentary leisure-time substitution and subgroups. In general, there was limited evidence for interaction when MI was modelled as the outcome, and no consistent evidence for effect modification by sex. There was evidence in short (<7 h/day) sleepers that the associations of reallocating sedentary leisure-time to sleep with diabetes and stroke were stronger in younger and middle-aged adults compared to older adults aged 60–80 years at baseline (*p*-interaction ≤0.001). With few exceptions, the associations of reallocating sedentary leisure-time to active behaviours with diabetes and stroke were stronger in participants with central obesity compared to those without central obesity (*p*-interaction ≤0.002). In addition, the associations of reallocating sedentary leisure-time to exercise behaviours were generally stronger in participants who were using antihypertensive medications compared to those who were not (*p*-interaction ≤0.068).

### Potential impact fractions

**Fig. 3:**
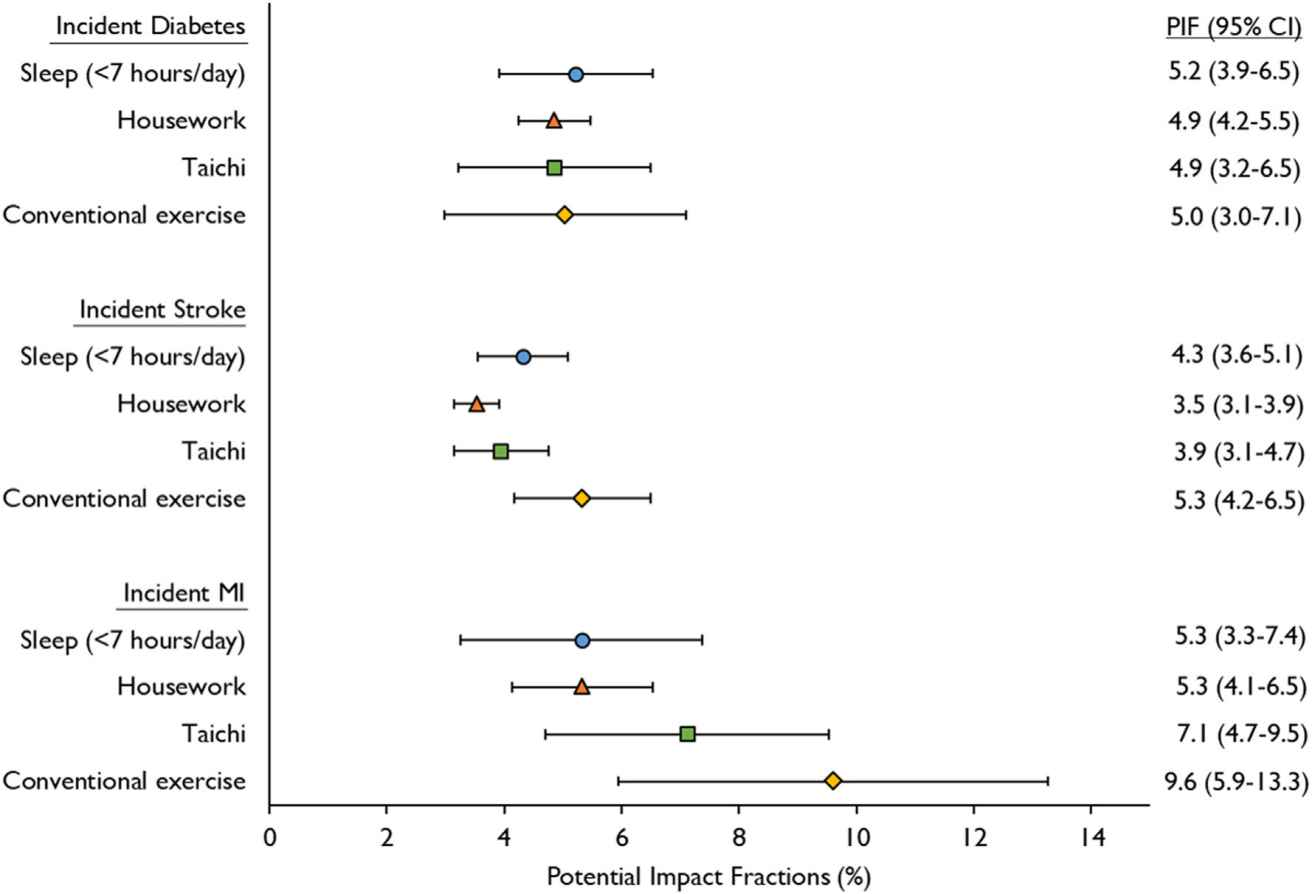
**The estimated decrease in incident cardiometabolic disease cases within the CKB cohort if average sedentary leisure-time was decreased by 30 min/day and the average time in each of the alternative discretionary movement behaviours was increased by the same amount, assuming causality**. Values are potential impact fractions (95% confidence interval) calculated using the ‘distribution shift’ method, based on a normal distribution for sedentary leisure-time, and hazard ratio estimates from Cox regression models using age as the underlying timescale and incorporating sex and region strata. Models adjusted for educational attainment, household income, work-related activity, smoking status, alcohol intake, daytime napping, healthy diet score, use of antihypertensive medication, family history of diabetes, heart attack or stroke, and total discretionary time-use. Potential impact fractions for reallocating sedentary leisure-time to sleep in longer (≥7 h/day) sleepers were not calculated as there was no evidence for associations (see Table 3), let alone causality. Abbreviation: MI, Myocardial Infarction.

Fig. 3 displays the estimated potential impact fractions associated with time reallocations. Assuming causality, if an average 30 min/day of sedentary leisure-time was replaced by the same amount of time in any alternative movement behaviour (except sleep in longer sleepers), it is estimated that approximately 5% of incident diabetes cases may have been averted in the CKB cohort (for example, the potential impact fraction [95% confidence interval] associated with reallocating sedentary leisure-time to housework was 4.9% [4.2–5.5%]). Replacing sedentary leisure-time with sleep (in short sleepers), housework or Taichi was associated with a lower proportion of incident stroke and MI cases, and the largest associations were observed when time was displaced to conventional exercise. If 30 min/day of sedentary leisure-time was replaced by conventional exercise, it is estimated that 5.3% (4.2–6.5%) and 9.6% (5.9–13.3%) of incident stroke and MI cases may have been averted in the CKB cohort, respectively.

### Sensitivity analysis

The results of the sensitivity analyses were generally similar to those of the main analysis (Table S3). The time-substitution results differed only minimally upon progressive adjustment for confounders, but adjusting for waist circumference diminished the beneficial associations of reallocating sedentary leisure-time to active behaviours on diabetes risk (Table S4).

## Discussion

This study investigated the potential for preventing common cardiometabolic diseases through replacement of sedentary leisure-time with non-sedentary discretionary behaviours in a large sample of Chinese adults. We found that sedentary leisure-time replaced by any non-sedentary behaviour (except sleep in longer sleepers) was associated with lower risks of diabetes, stroke and MI.

### Substitution of sedentary leisure-time with active behaviours

To our knowledge, only one previous study of Chinese middle-aged adults (*n* = 93,100) has incorporated isotemporal substitution methodology to quantify the associations of reallocating sedentary time to physical activity on incident cardiovascular diseases. In that study, among participants who reported ≥5 h/day of total daily sedentary time, substituting sedentary time with intensity-specific physical activity was associated with lower disease risk, with higher intensity physical activities yielding stronger associations.^20^ We also found a relatively larger effect estimate for stroke and particularly MI risk when sedentary leisure-time was replaced by conventional exercise rather than less intense activities. This may be because conventional exercise is usually performed with higher intensity and consequently higher energy expenditure for a given duration. Our findings for physical activity relative to diabetes risk are in line with the results of two prospective cohort studies of middle-aged and elderly men and women living in urban Shanghai. In non-isotemporal analyses, the authors concluded that daily life activities (including walking, stair climbing, bicycling, and housework), leisure-time exercise and sports were each associated with approximately equally lower risks of diabetes.^45^^,^^46^ Our results emphasise that replacement of sedentary leisure-time with any non-sedentary behaviour is associated with a lower risk of cardiometabolic diseases in Chinese adults. Several mechanisms may explain these associations including favourable influences upon major intermediate cardiometabolic risk factors such as adiposity, blood pressure, circulating glucose, lipid and inflammatory biomarkers.^47^, ^48^, ^49^

### Substitution of sedentary leisure-time with sleep

A non-isotemporal analysis of CKB data recently identified U-shaped associations between sleep duration with the risk of stroke and coronary heart disease, with individuals reporting 7–8 h/day of sleep having the lowest risks.^29^ A small number of prospective cohort studies have likewise reported that short sleep duration is associated with a higher risk of cardiovascular events in Chinese adults, including diabetes.^22^, ^23^, ^24^ In the current study, by incorporating isotemporal substitution methodology and sleep as a piecewise variable, we have demonstrated that reallocating sedentary leisure-time to sleep is favourably associated with cardiometabolic diseases in short (<7 h/day) but not in longer (≥7 h/day) sleeping Chinese adults. Isotemporal studies of UK and Australian adults have previously reported that shifting sedentary time to sleep in short sleepers was beneficial for incident diabetes and all-cause mortality risk.^30^^,^^31^ Our findings are also consistent with Mendelian randomisation studies, which have indicated that genetically predicted short (but not long) sleep duration is a potential causal risk factor for several cardiometabolic diseases.^50^^,^^51^

### Implications

Our results support Chinese physical activity guidelines which acknowledge the importance of less sedentary time and regular physical activity,^14^ and a central objective of the *Healthy China 2030* action plan, which is to increase the number of individuals who engage in regular exercise of at least moderate intensity.^16^ Indeed, we observed that reallocating sedentary leisure-time to conventional exercise was associated with the most marked reductions in cardiometabolic disease risk. It should be considered, however, that most effective behaviour change interventions tend to achieve displacement of sedentary time to lighter intensity physical activities, whereas increases in moderate-to-vigorous intensity physical activity are setting-specific and difficult to achieve and maintain in practice.^18^^,^^52^ Notably, we estimate that between 3.5% and 7% of incident cardiometabolic disease cases could have been averted in the CKB cohort if 30 min/day of sedentary leisure-time were replaced by either housework or Taichi. These behaviours may be more easily adopted by adults who are not initially willing or able to perform structured sports and exercise. Policy makers may consider broadening the scope of future action plans and physical activity guidelines to include the recommendation of multiple accessible types of health-enhancing physical activity including gentle forms of exercise (i.e., Taichi) and housework, alongside conventional exercise.

With regard to sleep duration, approximately 4% of incident stroke cases, and 5% of diabetes and MI cases, may have been averted in the CKB cohort if 30 min/day of sedentary leisure-time had been replaced by sleep in short (<7 h/day) sleepers. This particular finding ratifies another aim of the *Healthy China 2030* action plan, which is to increase the average sleep duration of adults to 7–8 h/day.^16^ We suggest that this goal may be achieved via a dual policy that emphasises reduced sedentary leisure-time alongside longer sleep, and which specifically targets adults who are failing to meet the minimum recommended sleep duration of 7 h/day. The results of our interaction analyses further highlight that particular sub-groups of the Chinese population may benefit more from efforts to replace sedentary time with sleep (younger and middle-aged adults) or physical activity (adults with central obesity or using antihypertensive medications).

### Strengths and weaknesses

We quantified the lower incidence of cardiometabolic diseases associated with exchanging sedentary leisure-time for alternative discretionary movement behaviours in a large, well-defined sample of Chinese adults. We focussed on sedentary leisure-time which is amenable to change through intervention,^18^ and examined replacement behaviours within the home and leisure domains (including sleep) as they reflect the most likely and realistic behaviour change scenarios when displacing sedentary leisure-time. The movement behaviour data were self-reported using questions that have not been directly examined for criterion validity, but they were at least adapted from validated instruments.^27^^,^^28^ Another limitation is that more detailed information about the activity intensity and types of non-sedentary movement behaviours performed was not available, and that the present analysis included only baseline movement behaviour data. A combination of measurement error and within-person variability means that relying upon a single measure of movement behaviours may have led to an underestimation of the disease risk associations.^53^ We adjusted for a diverse range of potential confounders including demographic, lifestyle, and medical factors, but there is scope for residual confounding as the data were self-reported and only available at the baseline survey. It is important to consider that our isotemporal substitutions are based upon theoretical not actual time reallocations. Furthermore, no firm causal conclusions can be made on account of the observational study design. The results require replication in other East Asian cohorts.

### Conclusion

Replacing sedentary leisure-time with non-sedentary behaviours including sleep (in short sleepers), housework, Taichi, and conventional exercise is associated with lower risks of common cardiometabolic diseases in Chinese adults. Prevention strategies should be developed to promote optimal levels of sleep and movement behaviours at the expense of sedentary leisure-time.

## Contributors

P.J.C. contributed to the conceptualisation of the study, data acquisition, the statistical analysis, and drafted an initial version of the manuscript. M.W. contributed to the conceptualisation of the study and data acquisition. H.H.S.H performed the statistical analysis. S.J.S. contributed to specification of the statistical analysis. Y.K. contributed to the conceptualisation of the study, data acquisition, and the specification of the statistical analysis. P.J.C and Y.K. directly accessed and verified the underlying data reported in the manuscript. All authors provided assistance with the interpretation of the study findings, critically reviewed a draft version of the manuscript for important intellectual content, and approved the version to be published.

## Data sharing statement

The China Kadoorie Biobank (CKB) is a global resource for the investigation of lifestyle, environmental, blood biochemical and genetic factors as determinants of common diseases. The CKB study group is committed to making the cohort data available to the scientific community in China, the UK and worldwide to advance knowledge about the causes, prevention and treatment of disease. For detailed information on what data is currently available to open access users and how to apply for it, visit: https://www.ckbiobank.org/data-access.

Researchers who are interested in obtaining the raw data from the China Kadoorie Biobank study that underlines this paper should contact ckbaccess@ndph.ox.ac.uk. A research proposal will be requested to ensure that any analysis is performed by bona fide researchers and–where data is not currently available to open access researchers–is restricted to the topic covered in this paper.

## Declaration of interests

The sponsors had no role in the design, analysis, interpretation or drafting of the manuscript. All authors declare that they have no competing interests in relation to publication of this report.
