# Supplementary material for: Associations of reallocating sedentary leisure-time to alternative discretionary movement behaviours with incident cardiometabolic diseases in 0.5 million Chinese adults
Source: Lancet Reg Health West Pac. 2025 Mar 23;57:101524. doi: 10.1016/j.lanwpc.2025.101524 (PMC11979937; doi:10.1016/j.lanwpc.2025.101524)
Supplement: Supplementary Tables [file mmc1.docx]

**Table S1. Associations of reallocating sedentary leisure time to alternative discretionary movement behaviours with incident cardiometabolic diseases, per 10 minutes/day time-substitution increments**

|  | **Incident Diabetes** (*n*=19,738) | **Incident Stroke** (*n*=51,460) | **Incident M**I (*n*=6,767) |
| --- | --- | --- | --- |
| **a) Substitution of 10 minutes/day of sedentary leisure time** |  |  |  |
| Sleep |  |  |  |
| Short sleepers (<7 hours/day) | 0.990 (0.984-0.995) | 0.993 (0.990-0.996) | 0.989 (0.981-0.998) |
| Longer sleepers (≥7 hours/day) | 0.999 (0.996-1.002) | 1.002 (1.000-1.003) | 0.998 (0.993-1.002) |
| Housework | 0.991 (0.989-0.994) | 0.996 (0.995-0.998) | 0.989 (0.984-0.994) |
| Taichi / qigong | 0.991 (0.985-0.998) | 0.995 (0.991-0.998) | 0.982 (0.972-0.992) |
| Conventional exercise | 0.990 (0.982-0.998) | 0.989 (0.985-0.994) | 0.972 (0.958-0.986) |
| **b) Substitution of 20 minutes/day of sedentary leisure time** |  |  |  |
| Sleep |  |  |  |
| Short sleepers (<7 hours/day) | 0.979 (0.968-0.990) | 0.986 (0.980-0.993) | 0.978 (0.962-0.996) |
| Longer sleepers (≥7 hours/day) | 0.998 (0.992-1.003) | 1.003 (1.000-1.007) | 0.995 (0.986-1.005) |
| Housework | 0.982 (0.977-0.987) | 0.993 (0.989-0.996) | 0.978 (0.969-0.988) |
| Taichi / qigong | 0.982 (0.969-0.995) | 0.989 (0.983-0.996) | 0.964 (0.945-0.984) |
| Conventional exercise | 0.981 (0.965-0.997) | 0.978 (0.969-0.988) | 0.945 (0.918-0.973) |
| **c) Substitution of 30 minutes/day of sedentary leisure time** |  |  |  |
| Sleep |  |  |  |
| Short sleepers (<7 hours/day) | 0.969 (0.953-0.985) | 0.980 (0.970-0.989) | 0.968 (0.943-0.993) |
| Longer sleepers (≥7 hours/day) | 0.996 (0.988-1.005) | 1.005 (0.999-1.010) | 0.993 (0.979-1.007) |
| Housework | 0.973 (0.966-0.981) | 0.989 (0.984-0.994) | 0.968 (0.954-0.981) |
| Taichi / qigong | 0.973 (0.954-0.993) | 0.984 (0.974-0.994) | 0.947 (0.919-0.976) |
| Conventional exercise | 0.971 (0.948-0.995) | 0.968 (0.954-0.981) | 0.918 (0.879-0.960) |
| **d) Substitution of 40 minutes/day of sedentary leisure time** |  |  |  |
| Sleep |  |  |  |
| Short sleepers (<7 hours/day) | 0.959 (0.938-0.980) | 0.973 (0.960-0.986) | 0.957 (0.925-0.991) |
| Longer sleepers (≥7 hours/day) | 0.995 (0.984-1.007) | 1.006 (0.999-1.013) | 0.991 (0.972-1.010) |
| Housework | 0.965 (0.955-0.975) | 0.985 (0.979-0.992) | 0.957 (0.939-0.975) |
| Taichi / qigong | 0.965 (0.940-0.990) | 0.979 (0.966-0.992) | 0.930 (0.893-0.968) |
| Conventional exercise | 0.962 (0.931-0.994) | 0.957 (0.940-0.975) | 0.893 (0.842-0.946) |
| **e) Substitution of 50 minutes/day of sedentary leisure time** |  |  |  |
| Sleep |  |  |  |
| Short sleepers (<7 hours/day) | 0.949 (0.923-0.976) | 0.966 (0.951-0.982) | 0.947 (0.907-0.989) |
| Longer sleepers (≥7 hours/day) | 0.994 (0.980-1.008) | 1.008 (0.999-1.017) | 0.988 (0.965-1.012) |
| Housework | 0.956 (0.944-0.968) | 0.982 (0.974-0.990) | 0.947 (0.925-0.969) |
| Taichi / qigong | 0.956 (0.925-0.988) | 0.974 (0.957-0.990) | 0.913 (0.868-0.960) |
| Conventional exercise | 0.952 (0.914-0.992) | 0.947 (0.925-0.969) | 0.868 (0.807-0.934) |
| **f) Substitution of 60 minutes/day of sedentary leisure time** |  |  |  |
| Sleep |  |  |  |
| Short sleepers (<7 hours/day) | 0.939 (0.908-0.971) | 0.960 (0.941-0.979) | 0.937 (0.889-0.987) |
| Longer sleepers (≥7 hours/day) | 0.993 (0.976-1.010) | 1.009 (0.999-1.020) | 0.986 (0.958-1.014) |
| Housework | 0.947 (0.933-0.962) | 0.978 (0.969-0.988) | 0.936 (0.910-0.963) |
| Taichi / qigong | 0.947 (0.911-0.985) | 0.969 (0.949-0.989) | 0.897 (0.844-0.952) |
| Conventional exercise | 0.943 (0.898-0.991) | 0.937 (0.911-0.963) | 0.844 (0.773-0.921) |

Values are hazard ratios (95% confidence interval) calculated from Cox regression models, using age as the underlying timescale, and incorporating sex and region strata. Models adjusted for educational attainment, household income, work-related activity, smoking status, alcohol intake, daytime napping, healthy diet score, use of antihypertensive medication, family history of diabetes, heart attack or stroke, and total discretionary time-use. Abbreviations: MI – Myocardial Infarction

**Table S2. Associations of reallocating sedentary leisure time to alternative discretionary movement behaviours with cardiometabolic diseases stratified by a) sex, b) baseline age group, c) central obesity status, and d) antihypertensive medication use, per 30 minutes/day time exchanges**

|  | **Incident Diabetes** | ***p*-interaction** | **Incident Stroke** | ***p*-interaction** | **Incident MI** | ***p*-interaction** |
| --- | --- | --- | --- | --- | --- | --- |
| **a) Sex** |  |  |  |  |  |  |
| **Males** (*n*=189,601) | *n*=7,647 |  | *n*=23,525 |  | *n*=3,915 |  |
| Sleep |  |  |  |  |  |  |
| Short sleepers (<7 hours/day) | 0.968 (0.939-0.998) |  | 0.974 (0.958-0.989) |  | 0.956 (0.920-0.994) |  |
| Longer sleepers (≥7 hours/day) | 0.986 (0.973-0.999) |  | 1.015 (1.008-1.023) |  | 0.994 (0.976-1.013) |  |
| Housework | 0.959 (0.946-0.973) |  | 0.994 (0.986-1.001) |  | 0.970 (0.952-0.989) |  |
| Taichi / qigong | 0.962 (0.932-0.992) |  | 0.985 (0.971-1.000) |  | 0.956 (0.920-0.994) |  |
| Conventional exercise | 0.952 (0.915-0.991) |  | 0.966 (0.946-0.986) |  | 0.913 (0.863-0.967) |  |
| **Females** (*n*=272,769) | *n*=12,091 |  | *n*=27,935 |  | *n*=2,852 |  |
| Sleep |  |  |  |  |  |  |
| Short sleepers (<7 hours/day) | 0.971 (0.952-0.991) | 0.07 | 0.984 (0.972-0.996) | 0.63 | 0.986 (0.951-1.021) | 0.08 |
| Longer sleepers (≥7 hours/day) | 1.003 (0.992-1.014) | 0.32 | 0.995 (0.988-1.002) | 0.001 | 0.990 (0.968-1.013) | 0.05 |
| Housework | 0.980 (0.970-0.989) | <0.001 | 0.986 (0.980-0.993) | 0.91 | 0.968 (0.948-0.988) | 0.67 |
| Taichi / qigong | 0.982 (0.957-1.007) | 0.11 | 0.982 (0.969-0.996) | 0.75 | 0.935 (0.891-0.980) | 0.27 |
| Conventional exercise | 0.984 (0.953-1.015) | 0.25 | 0.970 (0.952-0.989) | 0.50 | 0.927 (0.866-0.993) | 0.61 |
| **b) Baseline age group** |  |  |  |  |  |  |
| **Younger adults (aged 30 to <50 years)** (*n*=221,806) | *n*=6,649 |  | *n*=10,496 |  | *n*=1,029 |  |
| Sleep |  |  |  |  |  |  |
| Short sleepers (<7 hours/day) | 0.947 (0.912-0.984) |  | 0.942 (0.917-0.967) |  | 0.929 (0.847-1.019) |  |
| Longer sleepers (≥7 hours/day) | 0.988 (0.974-1.003) |  | 0.992 (0.980-1.004) |  | 0.990 (0.953-1.029) |  |
| Housework | 0.975 (0.961-0.989) |  | 0.990 (0.979-1.002) |  | 0.984 (0.945-1.025) |  |
| Taichi / qigong | 1.027 (0.978-1.078) |  | 1.005 (0.969-1.041) |  | 0.935 (0.810-1.078) |  |
| Conventional exercise | 0.988 (0.937-1.042) |  | 0.928 (0.888-0.970) |  | 0.932 (0.803-1.081) |  |
| **Middle-aged adults (aged 50 to <60 years)** (*n*=140,650) | *n*=7,396 |  | *n*=17,126 |  | *n*=1,782 |  |
| Sleep |  |  |  |  |  |  |
| Short sleepers (<7 hours/day) | 0.947 (0.922-0.972) |  | 0.958 (0.942-0.975) |  | 0.987 (0.929-1.049) |  |
| Longer sleepers (≥7 hours/day) | 0.995 (0.982-1.010) |  | 1.002 (0.993-1.011) |  | 0.986 (0.958-1.015) |  |
| Housework | 0.975 (0.963-0.986) |  | 0.985 (0.977-0.993) |  | 0.979 (0.952-1.006) |  |
| Taichi / qigong | 0.969 (0.938-1.001) |  | 0.996 (0.977-1.015) |  | 0.953 (0.891-1.018) |  |
| Conventional exercise | 0.973 (0.936-1.010) |  | 0.965 (0.942-0.989) |  | 0.904 (0.827-0.988) |  |
| **Older adults (aged 60 to <80 years)** (*n*=99,914) | *n*=5,693 |  | *n*=23,838 |  | *n*=3,956 |  |
| Sleep |  |  |  |  |  |  |
| Short sleepers (<7 hours/day) | 0.991 (0.965-1.017) | 0.001 | 0.995 (0.982-1.008) | <0.001 | 0.965 (0.936-0.995) | 0.49 |
| Longer sleepers (≥7 hours/day) | 1.003 (0.988-1.018) | 0.01 | 1.010 (1.003-1.018) | <0.001 | 0.995 (0.977-1.013) | 0.82 |
| Housework | 0.978 (0.964-0.992) | 0.79 | 0.992 (0.985-0.999) | 0.04 | 0.960 (0.943-0.977) | 0.31 |
| Taichi / qigong | 0.988 (0.960-1.017) | 0.03 | 0.988 (0.975-1.001) | 0.08 | 0.950 (0.917-0.984) | 0.95 |
| Conventional exercise | 0.976 (0.937-1.017) | 0.72 | 0.982 (0.964-1.000) | 0.03 | 0.927 (0.879-0.978) | 0.74 |
| **c) Central obesity status** |  |  |  |  |  |  |
| **No central obesity** (*n*=359,543) | *n*=11,109 |  | *n*=36,095 |  | *n*=4,721 |  |
| Sleep |  |  |  |  |  |  |
| Short sleepers (<7 hours/day) | 0.969 (0.948-0.990) |  | 0.984 (0.973-0.995) |  | 0.973 (0.944-1.003) |  |
| Longer sleepers (≥7 hours/day) | 1.004 (0.993-1.016) |  | 1.008 (1.002-1.014) |  | 0.985 (0.968-1.002) |  |
| Housework | 0.991 (0.981-1.001) |  | 0.996 (0.990-1.002) |  | 0.967 (0.951-0.984) |  |
| Taichi / qigong | 0.986 (0.959-1.014) |  | 0.994 (0.981-1.007) |  | 0.928 (0.891-0.965) |  |
| Conventional exercise | 0.979 (0.945-1.013) |  | 0.984 (0.967-1.001) |  | 0.932 (0.883-0.984) |  |
| **Central obesity** (*n*=102,827) | *n*=8,629 |  | *n*=15,365 |  | *n*=2,046 |  |
| Sleep |  |  |  |  |  |  |
| Short sleepers (<7 hours/day) | 0.970 (0.945-0.996) | 0.64 | 0.971 (0.953-0.989) | 0.65 | 0.956 (0.909-1.005) | 0.75 |
| Longer sleepers (≥7 hours/day) | 1.002 (0.990-1.015) | 0.37 | 1.002 (0.992-1.011) | 0.75 | 1.016 (0.990-1.042) | 0.03 |
| Housework | 0.972 (0.961-0.983) | <0.001 | 0.979 (0.971-0.988) | <0.001 | 0.976 (0.952-1.001) | 0.14 |
| Taichi / qigong | 0.971 (0.945-0.997) | <0.001 | 0.969 (0.953-0.986) | <0.001 | 0.975 (0.931-1.022) | 0.56 |
| Conventional exercise | 0.998 (0.963-1.033) | 0.22 | 0.944 (0.921-0.968) | 0.002 | 0.900 (0.835-0.970) | 0.10 |
| **d) Antihypertensive medication use** |  |  |  |  |  |  |
| **No** (*n*=429,868) | *n*=16,549 |  | *n*=43,523 |  | *n*=5,650 |  |
| Sleep |  |  |  |  |  |  |
| Short sleepers (<7 hours/day) | 0.971 (0.953-0.988) |  | 0.977 (0.967-0.988) |  | 0.960 (0.933-0.987) |  |
| Longer sleepers (≥7 hours/day) | 0.998 (0.988-1.007) |  | 1.002 (0.997-1.008) |  | 0.995 (0.979-1.010) |  |
| Housework | 0.975 (0.967-0.983) |  | 0.987 (0.981-0.992) |  | 0.965 (0.950-0.980) |  |
| Taichi / qigong | 0.978 (0.957-0.999) |  | 0.987 (0.976-0.998) |  | 0.951 (0.920-0.984) |  |
| Conventional exercise | 0.974 (0.948-1.001) |  | 0.968 (0.954-0.983) |  | 0.920 (0.876-0.966) |  |
| **Yes** (*n*=32,502) | *n*=3,189 |  | *n*=7,937 |  | *n*=1,117 |  |
| Sleep |  |  |  |  |  |  |
| Short sleepers (<7 hours/day) | 0.962 (0.923-1.003) | 0.55 | 0.992 (0.967-1.017) | 0.14 | 1.008 (0.943-1.077) | 0.49 |
| Longer sleepers (≥7 hours/day) | 0.990 (0.970-1.010) | 0.71 | 1.015 (1.002-1.029) | <0.001 | 0.986 (0.952-1.021) | 0.18 |
| Housework | 0.967 (0.970-0.985) | 0.38 | 0.998 (0.986-1.011) | 0.53 | 0.980 (0.947-1.015) | 0.95 |
| Taichi / qigong | 0.963 (0.923-1.005) | 0.001 | 0.984 (0.961-1.008) | <0.001 | 0.934 (0.874-0.997) | 0.22 |
| Conventional exercise | 0.963 (0.910-1.019) | 0.068 | 0.967 (0.935-1.001) | 0.004 | 0.923 (0.838-1.016) | 0.83 |

Values are hazard ratios (95% confidence interval) calculated from Cox regression models, using age as the underlying timescale, and incorporating sex and region strata. Models adjusted for educational attainment, household income, work-related activity, smoking status, alcohol intake, daytime napping, healthy diet score, use of antihypertensive medication, family history of diabetes, heart attack or stroke, and total discretionary time-use. To test for sex interaction, sex strata were removed, and sex was included in the linear predictor. To test for interaction by age group, baseline age group was included in the linear predictor. To test for interaction by obesity status, central obesity status was included in the linear predictor. Likelihood ratio tests were used to test for improved model fit by comparing models with and without the relevant interaction terms (e.g. time-substitution parameter*sex). Abbreviations: MI – Myocardial Infarction

**Table S3. Associations of reallocating sedentary leisure time to alternative discretionary movement behaviours with incident cardiometabolic diseases, per 30 minutes/day time exchanges, after excluding a) disease cases diagnosed within the first three years of follow-up, b) participants with poor self-rated general health at baseline, and c) participants with major prior chronic diseases**

|  | **Incident Diabetes** | **Incident Stroke** | **Incident MI** |
| --- | --- | --- | --- |
| **Whole sample results** (*n*=462,370) | *n*=19,738 | *n*=51,460 | *n*=6,767 |
| Sleep |  |  |  |
| Short sleepers (<7 hours/day) | 0.969 (0.953 to 0.985) | 0.980 (0.970 to 0.989) | 0.968 (0.943 to 0.993) |
| Longer sleepers (≥7 hours/day) | 0.996 (0.988 to 1.005) | 1.005 (0.999 to 1.010) | 0.993 (0.979 to 1.007) |
| Housework | 0.973 (0.966 to 0.981) | 0.989 (0.984 to 0.994) | 0.968 (0.954 to 0.981) |
| Taichi / qigong | 0.973 (0.954 to 0.993) | 0.984 (0.974 to 0.994) | 0.947 (0.919 to 0.976) |
| Conventional exercise | 0.971 (0.948 to 0.995) | 0.968 (0.954 to 0.981) | 0.918 (0.879 to 0.960) |
| **a) Excluding cases diagnosed within the first three years of follow-up** (*n*=452,248) | *n*=16,828 | *n*=44,096 | *n*=5,618 |
| Sleep |  |  |  |
| Short sleepers (<7 hours/day) | 0.974 (0.957 to 0.992) | 0.989 (0.979 to 1.000) | 0.975 (0.948 to 1.003) |
| Longer sleepers (≥7 hours/day) | 0.997 (0.988 to 1.006) | 1.007 (1.001 to 1.013) | 0.998 (0.983 to 1.014) |
| Housework | 0.964 (0.956 to 0.972) | 0.993 (0.988 to 0.998) | 0.975 (0.960 to 0.990) |
| Taichi / qigong | 0.962 (0.941 to 0.982) | 0.975 (0.964 to 0.986) | 0.930 (0.898 to 0.962) |
| Conventional exercise | 0.967 (0.942 to 0.993) | 0.969 (0.954 to 0.984) | 0.925 (0.882 to 0.971) |
| **b) Excluding participants with poor self-rated health at baseline** (*n*=421,236) | *n*=17,577 | n=45,457 | *n*=5,843 |
| Sleep |  |  |  |
| Short sleepers (<7 hours/day) | 0.983 (0.964-1.002) | 0.980 (0.970 to 0.991) | 0.970 (0.941 to 1.000) |
| Longer sleepers (≥7 hours/day) | 0.999 (0.990 to 1.008) | 1.004 (0.999 to 1.010) | 0.990 (0.975 to 1.006) |
| Housework | 0.974 (0.966 to 0.982) | 0.990 (0.985 to 0.995) | 0.970 (0.955 to 0.985) |
| Taichi / qigong | 0.978 (0.958 to 0.998) | 0.987 (0.976 to 0.998) | 0.947 (0.917 to 0.978) |
| Conventional exercise | 0.975 (0.950 to 0.999) | 0.970 (0.956 to 0.985) | 0.921 (0.880 to 0.964) |
| **c) Excluding participants with major prior chronic diseases** (*n*=407,411) | *n*=16,947 | *n*=43,152 | *n*=5,505 |
| Sleep |  |  |  |
| Short sleepers (<7 hours/day) | 0.973 (0.954 to 0.992) | 0.982 (0.971 to 0.993) | 0.962 (0.934 to 0.992) |
| Longer sleepers (≥7 hours/day) | 0.998 (0.989 to 1.007) | 1.002 (0.996 to 1.008) | 0.991 (0.975 to 1.007) |
| Housework | 0.972 (0.964 to 0.980) | 0.988 (0.983 to 0.993) | 0.972 (0.957 to 0.987) |
| Taichi / qigong | 0.978 (0.958 to 0.999) | 0.980 (0.969 to 0.991) | 0.936 (0.904 to 0.968) |
| Conventional exercise | 0.968 (0.942 to 0.994) | 0.962 (0.947 to 0.977) | 0.918 (0.875 to 0.963) |

Values are hazard ratios (95% confidence interval) calculated from Cox regression models, using age as the underlying timescale, and incorporating sex and region strata. Models adjusted for educational attainment, household income, work-related activity, smoking status, alcohol intake, daytime napping, healthy diet score, use of antihypertensive medication, family history of diabetes, heart attack or stroke, and total discretionary time-use. Abbreviations: MI – Myocardial Infarction

**Table S4. Associations of reallocating sedentary leisure time to alternative discretionary movement behaviours with incident cardiometabolic diseases following sequential adjustment for confounders and waist circumference, per 30 minutes/day time exchanges**

|  | **Incident Diabetes**  (*n*=19,738) | **Incident Stroke**  (*n*=51,460) | **Incident M**I  (*n*=6,767) |
| --- | --- | --- | --- |
| **a) Unadjusted** |  |  |  |
| Sleep |  |  |  |
| Short sleepers (<7 hours/day) | 0.969 (0.953-0.986) | 0.976 (0.967-0.986) | 0.961 (0.97-0.987) |
| Longer sleepers (≥7 hours/day) | 0.998 (0.990-1.006) | 1.005 (0.999-1.010) | 0.991 (0.977-1.005) |
| Housework | 0.969 (0.962-0.976) | 0.986 (0.982-0.991) | 0.961 (0.948-0.975) |
| Taichi / qigong | 0.986 (0.967-1.005) | 0.988 (0.979-0.998) | 0.953 (0.925-0.982) |
| Conventional exercise | 0.979 (0.956-1.003) | 0.963 (0.950-0.976) | 0.916 (0.877-0.956) |
| **b) Adjusted for educational attainment and household income** |  |  |  |
| Sleep |  |  |  |
| Short sleepers (<7 hours/day) | 0.968 (0.952-0.984) | 0.977 (0.967-0.986) | 0.962 (0.937-0.987) |
| Longer sleepers (≥7 hours/day) | 1.000 (0.991-1.008) | 1.005 (0.999-1.010) | 0.991 (0.977-1.005) |
| Housework | 0.970 (0.963-0.978) | 0.986 (0.981-0.990) | 0.961 (0.948-0.975) |
| Taichi / qigong | 0.983 (0.964-1.002) | 0.989 (0.979-0.999) | 0.954 (0.926-0.983) |
| Conventional exercise | 0.974 (0.951-0.998) | 0.965 (0.952-0.978) | 0.918 (0.879-0.958) |
| **c) Further adjusted for work-related activity, smoking status, alcohol intake, daytime napping, healthy diet score** |  |  |  |
| Sleep |  |  |  |
| Short sleepers (<7 hours/day) | 0.966 (0.950-0.982) | 0.977 (0.968-0.987) | 0.966 (0.942-0.992) |
| Longer sleepers (≥7 hours/day) | 0.995 (0.987-1.004) | 1.003 (0.998-1.008) | 0.992 (0.978-1.006) |
| Housework | 0.971 (0.964-0.979) | 0.986 (0.982-0.991) | 0.965 (0.951-0.978) |
| Taichi / qigong | 0.984 (0.965-1.003) | 0.993 (0.983-1.003) | 0.956 (0.928-0.985) |
| Conventional exercise | 0.977 (0.954-1.002) | 0.972 (0.958-0.985) | 0.924 (0.885-0.966) |
| **d) Further adjusted for use of antihypertensive medication, family history of diabetes, heart attack or stroke** |  |  |  |
| Sleep |  |  |  |
| Short sleepers (<7 hours/day) | 0.969 (0.953-0.985) | 0.980 (0.970-0.989) | 0.968 (0.943-0.993) |
| Longer sleepers (≥7 hours/day) | 0.996 (0.988-1.005) | 1.005 (0.999-1.010) | 0.993 (0.979-1.007) |
| Housework | 0.973 (0.966-0.981) | 0.989 (0.984-0.994) | 0.968 (0.954-0.981) |
| Taichi / qigong | 0.973 (0.954-0.993) | 0.984 (0.974-0.994) | 0.947 (0.919-0.976) |
| Conventional exercise | 0.971 (0.948-0.995) | 0.968 (0.954-0.981) | 0.918 (0.879-0.960) |
| **e) Further adjusted for waist circumference** |  |  |  |
| Sleep |  |  |  |
| Short sleepers (<7 hours/day) | 0.968 (0.952-0.984) | 0.980 (0.970-0.989) | 0.968 (0.943-0.993) |
| Longer sleepers (≥7 hours/day) | 1.008 (0.999-1.016) | 1.006 (1.001-1.012) | 0.995 (0.981-1.009) |
| Housework | 0.989 (0.981-0.996) | 0.992 (0.987-0.997) | 0.972 (0.958-0.985) |
| Taichi / qigong | 0.980 (0.961-0.999) | 0.985 (0.975-0.995) | 0.947 (0.919-0.976) |
| Conventional exercise | 0.999 (0.975-1.024) | 0.971 (0.958-0.985) | 0.921 (0.882-0.963) |

Values are hazard ratios (95% confidence interval) calculated from Cox regression models, using age as the underlying timescale, and incorporating sex and region strata. Abbreviations: MI – Myocardial Infarction
